# The Utility of Recording Submental Electrical Activity in Polysomnography

**DOI:** 10.7759/cureus.17216

**Published:** 2021-08-16

**Authors:** Muhammad Najjar

**Affiliations:** 1 Internal Medicine, University of Illinois at Chicago, Chicago, USA

**Keywords:** polysomnography, muscle tonus, electromyography, sleep apnea, obstructive, central, artifact

## Abstract

Background

Polysomnography is widely utilized as a diagnostic tool in sleep medicine. It is used to diagnose a variety of sleep disorders, including obstructive and central sleep apnea, periodic limb movements, parasomnia, and other sleep disorders. The objective of this study is to discuss the utility of recording submental electrical activity as a tool that provides the additional information needed in producing accurate results of a polysomnogram and emphasize the importance of including this recording in home sleep apnea tests.

Methods

A total of 1472 consecutive polysomnograms were used to assess the usefulness of submental activity in contributing to the accurate reporting of polysomnographic findings. These records were gathered over a one-year period at a major academic sleep center.

Results

Some of the observed electrical activities in the submental area are rare, although they may be important in confirming the findings of the polysomnogram or provide additional information. Adding an electroencephalographic channel and submental electromyographic recording is likely to increase the accuracy of home sleep apnea tests.

Conclusions

Recording of submental electrical activity is an integral part of polysomnography and provides more information than just recording the muscle tone of the submental area and is important in increasing the accuracy of home sleep apnea tests.

## Introduction

Polysomnography (PSG) is an important diagnostic tool in sleep medicine. A polysomnogram needs to be performed in most patients with sleep disorders apart from those who have typical insomnia. Polysomnography is still an important diagnostic tool when evaluating patients with sleep-disordered breathing although there is a recent tendency to use home-based unattended sleep apnea tests in the evaluation of obstructive sleep apnea (OSA).

Interest in home sleep apnea testing started in the 1990s to make access to sleep testing faster, easier, and less expensive. Early research focused mostly on the validation of home sleep apnea tests (HSATs) comparing the results of HSATs to PSGs [1-2]; more recently, interest shifted to assessing the quality of HSATs and optimizing their accuracy [3-4]. The use of HSATs was studied in subpopulations of patients such as those with chronic heart failure [5], stroke [6], and children [7]. Some investigators have recommended implementing strategies to improve and standardize the process of sampling patients for HSATs, address instrumentation challenges, data processing, clinical data reporting, and patient acceptability [8]. The American Academy of Sleep Medicine has also published a position statement regarding the clinical use of HSATs [9].

Polysomnography allows us to monitor multiple physiological functions simultaneously, a unique feature of this test. The ability to record various parameters, have this data available simultaneously, and compare the function of various physiological systems at the same time makes PSG a powerful and unique tool in assessing sleep disorders.

One feature of polysomnography is the redundancy in collecting data, this is, at times, helpful when an artifact obscures the ability to view data accurately from one or more channels, and sometimes, it adds an additional use or function for every channel used in the recording.

One of the functions recorded in polysomnography is muscle tone in the submental area; this is a recommended function based on the American Academy of Sleep Medicine manual for the scoring of sleep and associated events [10]. The manual recommends using surface electromyography (EMG) to record the activity of chin muscles. It is recommended that three electrodes be used: one is placed midline 1 cm above the lower edge of the mandible, and the other 2 are placed 2 cm below the inferior edge of the mandible 2 cm to the right and 2 cm to the left of midline. Only one channel is displayed using one of the electrodes below the mandible referred to the one above, the other electrode below the mandible is only used as a backup in case the other electrode malfunctions.

In this study, we describe a series of findings in the submental electrodes of PSG and review some of the benefits of monitoring the electrical activity recorded in the submental channel when interpreting a PSG. The main purpose of this study is to emphasize the importance of recording electrical activity from the submental area and that this should be incorporated even in abbreviated sleep studies that tend to record mainly respiratory parameters and not include electroencephalographic (EEG) or electromyographic (EMG) recordings.

A recent study has suggested that home monitoring systems include EEG and EMG to monitor sleep over multiple consecutive nights to avoid the need to perform PSGs, which tend to be expensive and inconvenient. The use of EMG data increased the usefulness and accuracy of these studies [4].

## Materials and methods

All polysomnograms performed between February 1, 2019, and January 31, 2020, were used for assessing the usefulness of submental electrical activity when analyzing polysomnographic findings especially for proper scoring. A total of 1472 polysomnograms were used for the study; they were all scored based on the recommended scoring rules of the American Academy of Sleep Medicine manual and reviewed by a sleep physician who is trained and certified in sleep medicine. All these studies were performed by certified PSG technicians.

All epochs of every sleep study were analyzed visually to determine their usefulness in scoring sleep stage, arousal, and abnormal activity as well as any other finding that is of value in interpreting the PSG.

The information we found in this paper emphasizes the importance of submental muscle tone monitoring and suggests that it may need to be included in home sleep apnea testing. We also draw attention to the importance of recording and assessing electrical activity in the submental area and reviewing it carefully due to the valuable information it may provide.

## Results

In analyzing the usefulness of submental electrical activity in PSGs, we found that information obtained from analyzing this activity is important in seven sets of categories.

Scoring a sleep stage

Monitoring chin tone was needed for proper scoring of sleep stage in 1472 of 1472 polysomnograms, that is, 100%. It was not needed for all epochs but was needed in 148408 epochs, virtually for scoring every epoch of rapid eye movement (REM) sleep. In REM, sleep chin tone should the lowest or at least as low as the lowest recorded in any other sleep stage.

Scoring arousals

The activity of chin tone was needed to score arousals in 33,391 epochs, that is, all epochs of REM sleep and 11,130 epochs of NREM sleep, which is approximately 4.5% of all epochs. To score arousal in REM sleep, chin tone must increase for at least one second. Surface EMG has advantages and disadvantages, some of its advantages include the easy application of EMG electrodes, the closeness of submental muscles to the electrodes, the ability to get needed data about chin tone easily, and avoiding the use of needles that cause pain but provide more reliable data that can be quantified, this data, however, is not needed for the purpose of standard PSG [11].

Evaluation of the activity of upper airway dilating muscles, snoring, and air leaks

**Figure 1:**
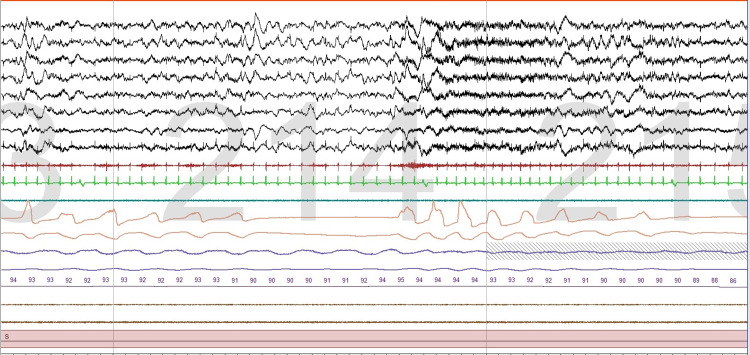
Obstructive apnea This one-minute epoch shows obstructive apnea with a reduction of the activity of upper airway dilator muscles followed by an arousal and increased muscle tone.

We found this to be important in 473 studies, that is, approximately 32% of all PSGs reviewed. Submental EMG sometimes allows the assessment of upper airway muscle activity thus providing useful information about the tone of upper airway dilating muscles. For example, the activity of these muscles is high in patients with severe OSA and patients with loud snoring [12] and is low during REM sleep, and in association with obstructive respiratory events in REM sleep [13], this provides a visual observation of why obstructive sleep apnea tends to be worse in REM sleep when muscle tone is low (Figure 1).

**Figure 2:**
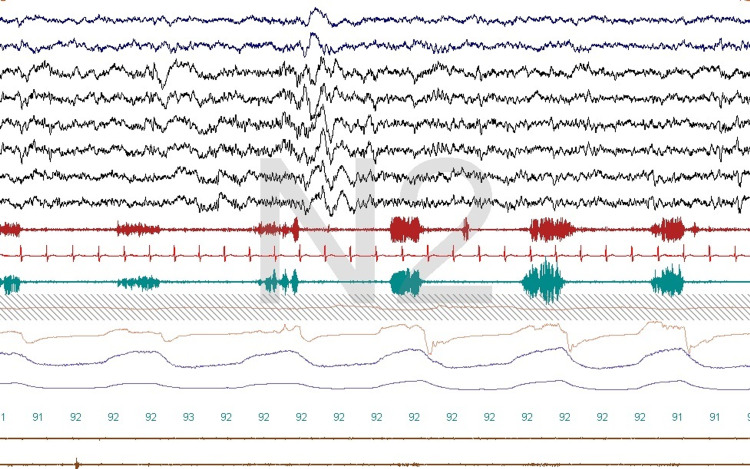
Snoring artifact In this 30-second epoch, snoring is clearly seen not only in the snore channel but is also picked up by submental electrodes as an artifact.

Submental electrodes may also pick up a snoring artifact when electrodes are not applied properly or if they move during sleep (Figure 2). Experience and skill are needed to differentiate the activity of upper airway dilator muscles from snoring artifacts, as both may be seen in the same setting. It is well-known that many patients with OSA are obese, and many have significant fat deposits below the mandible limiting the ability to record the activity of upper airway dilators, however, this is not always the case and sometimes, this activity can be observed easily. It may not be consistent all night long, as body position, electrode movement, or the pressure applied on the electrode vary during the recording.

**Figure 3:**
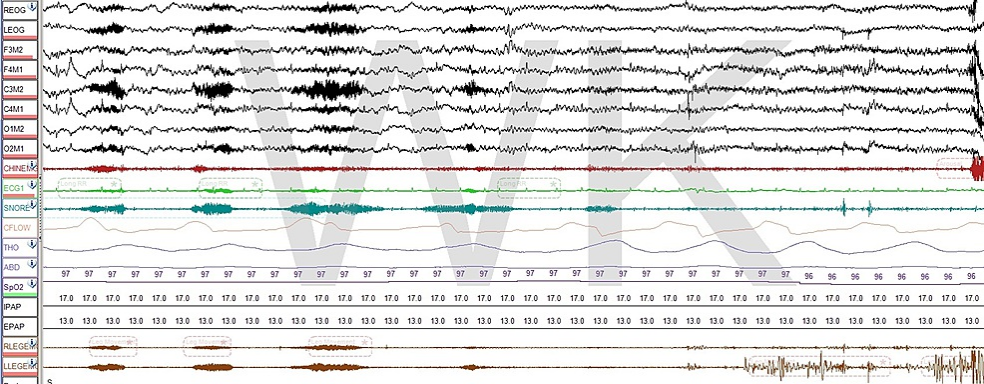
Air leak artifact A significant air leak artifact is observed in multiple channels, including the submental channel, in this 30-second epoch.

Another artifact that may also be observed in the submental area and occurs in the context of sleep apnea treatment is air leak artifact that occurs during positive airway pressure titration (Figure 3). It is obviously important for sleep technicians to recognize air leaks seen in PSGs and address them immediately.

Differentiation between obstructive and central events

**Figure 4:**
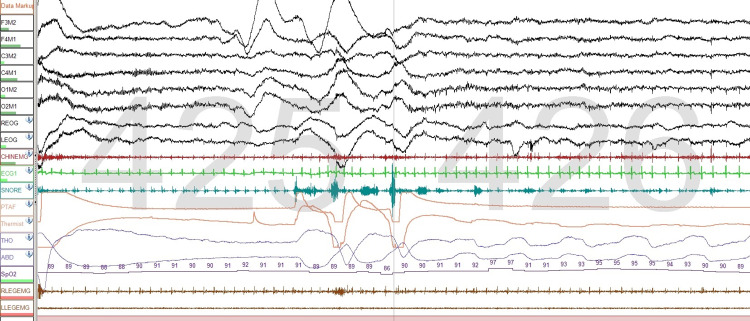
Central and obstructive sleep apnea A central apnea occurring after an arousal is seen followed by an obstructive event. There is no discernible activity in upper airway dilator muscles during the central event, and some activity is observed during the obstructive event. This montage is the same as in Figures 1, 2.

We found that chin tone reduction during central apnea was consistent, and we found this to be helpful in 43 polysomnograms, that is, about 3% of the time. Upper airway muscle activity is reduced or absent during central sleep apnea; this can be seen when all technical parameters are optimally met and can help differentiate obstructive from central events (Figure 4). It is thought that the respiratory center that controls breathing in the central nervous system provides simultaneous stimulation to both the diaphragm and chest wall muscles, that is, the pump muscles and the genioglossus and other upper airway dilators. This ensures airway patency during inhalation, and this obviously fails in patients with OSA [14].

Submental EMG is also important when assessing patients with REM sleep behavior disorder (RBD)

**Figure 5:**
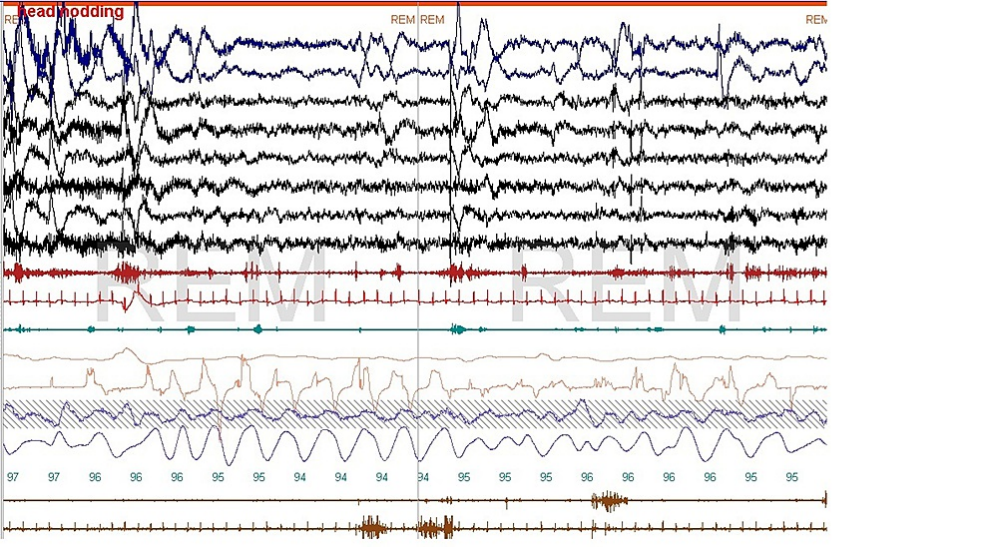
REM sleep behavior disorder A recording for a patient who has RDB. Submental muscle activity is clearly increased during an episode of dream-enactment arising in REM sleep. One-minute epoch The montage is the same as Figure 4.

In the sample we used, we found that in 19 sleep studies observing the increased activity of submental muscles was needed to score studies accurately. this was the case in approximately 1.3% of the PSGs evaluated. Normally, REM sleep is characterized by muscle atonia, however, in RBD this does not occur, and submental muscles show evidence of tonic activity, that is, increased muscle tone through much of an epoch or transiently increased muscle activity also in the majority of an epoch. It is during these periods when violent movements occur and patients have dreams of violence and act out these dreams (Figure 5). A more detailed assessment of chin tone using digital analysis of three-second segments has proven to be more valuable in diagnosing RBD than visual analysis of chin tone [15].

Recording bruxism

**Figure 6:**
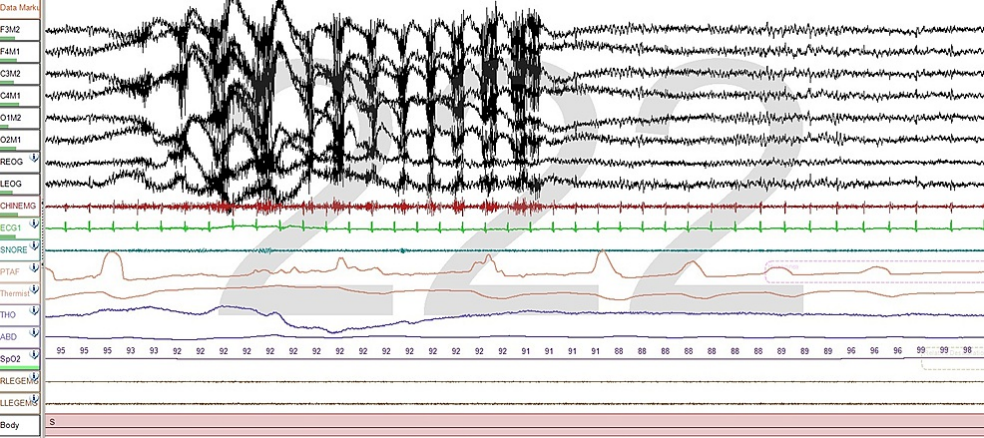
Bruxism There are changes in submental muscle tone along with electrical activity arising from temporal and masticatory muscles. Thirty-second epoch

We found that submental muscle activity was increased during periods of bruxism in 97 PSGs. The increase in chin tone was helpful in confirming bruxism; however, in only 20 studies. Muscle tone is increased as either a tonic or phasic phenomenon. The increase in muscle activity also affects temporal and masticatory muscles. The increase in the tone of submental muscles is typically observed during arousals (Figure 6).

Recording of sleep spindles

**Figure 7:**
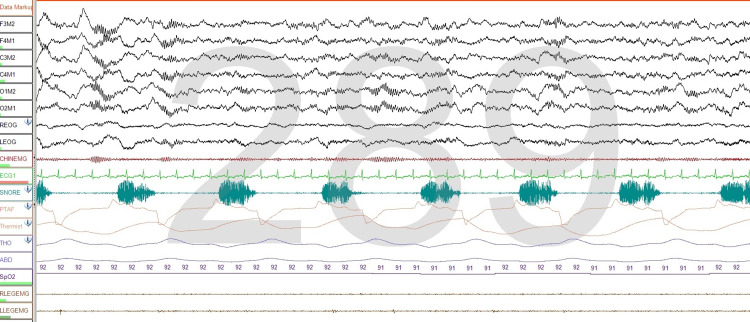
Sleep spindles In this 30-second epoch, sleep spindles are seen in the submental area. This is especially seen in patients taking benzodiazepines and may be misinterpreted as an increase in chin tone or arousal.

We found that chin electrodes surprisingly recorded sleep spindles in 28 studies. The surface electrodes placed in the submental area may pick up EEG activity, and this seems to be especially true for sleep spindles. This observation is not necessarily uncommon and is important to recognize for two reasons: it helps in scoring an epoch as stage 2 sleep if the scalp EEG electrodes malfunction or an artifact obscures observing their activity and more importantly to avoid scoring arousals if sleep spindles are mistakenly thought to represent increased muscle activity associated with an arousal (Figure 7).

We also found that electrocardiographic (EKG) activity may also be seen in chin electrodes, and although EKG is easily recorded using chest, arm, or leg electrodes, it is occasionally helpful to see it in other electrodes if EKG electrodes malfunction.

There are also reports in the literature of other advantages of observing what electrodes in the submental area may display. A report, for example, showed that these electrodes may pick up artifacts of vagal nerve stimulator activity used for the treatment of patients who have epilepsy [16].

## Discussion

Recording electromyographic activity in the submental region is an essential component in polysomnography. Traditionally, the data recorded from this area provide information about muscle tone in the submental area. This information is important for scoring sleep stage, especially REM sleep, as well as scoring electrographic arousals occurring in REM sleep.

It is not always easy to differentiate obstructive from central sleep apnea [17] and although the information provided by submental EMG is not always essential in differentiating between obstructive and central sleep apnea, careful observation suggests that it may be helpful in differentiating between them. It is important to keep in mind that electromyographic activity in the submental region does not reflect the activity of a single muscle, rather it reflects the activity of multiple muscles in the submental region [18].

There is a tendency to use home sleep apnea tests (HSAT) to diagnose obstructive sleep apnea. One of the limitations of HSAT is the inability to determine total sleep time, and as a result, the calculated respiratory event index may be inaccurate. Some researchers have suggested the use of frontal electroencephalography along with submental electromyography to score sleep and its various stages. This was shown to be consistent with standard polysomnography [19] and highlights the importance of recording submental EMG in accurately scoring sleep and increasing the accuracy of the calculated apnea-hypopnea index recorded in HSAT when frontal EEG along with submental EMG are used as additional channels [20].

Multiple factors predispose to OSA, including the tone of the upper airway dilating muscles, abnormal anatomy, obesity, family history, genetic predisposition, as well as hormonal factors [21]. There is considerable interest in trying to increase the muscle tone of upper airway dilating muscles to treat OSA. Clinical trials are underway to test the effectiveness of α-adrenergic stimulation and acetylcholine muscarinic antagonism as pharmacological treatments for OSA. Although it is unlikely that standard polysomnography will show increased muscle tone in the submental area due to drug therapy for OSA; the future will tell if this will be the case or not [22].

Monitoring submental muscle tone does not only have diagnostic implications. Recent studies suggest that analyzing the activity of the genioglossus muscle may also have therapeutic implications, as patients with physiological abnormalities of the upper airway are less likely to respond to surgical intervention for the treatment of obstructive sleep apnea [23].

The electrical activity recorded in the submental area is not only generated by muscles. Electrodes may also pick up electrocardiographic or EEG activity such as sleep spindles. The ability of the chin electrodes to record EEG activity is determined by the location of these electrodes and their filter setting. The low-frequency filter for chin muscle tone recording is usually set at 10 Hz, and the high-frequency filter is set at 100 Hz, meaning that these electrodes will not display slow waves of sleep such as delta waves or K complexes but will show sleep-related EEG activity of fast frequency, provided that the generator of this activity is close to the electrodes.

Sleep spindles are generated by the reticular thalamic nucleus and are best recorded in the central area of the scalp [24]; however, their distribution can be widespread, and they can be recorded in the submental area. It is important to avoid erroneously scoring sleep spindles as arousals especially in the case of a patient who is on benzodiazepines.

In summary, monitoring muscle tone using submental electrodes is an essential feature of a PSG, and although these electrodes are important in scoring sleep stages and REM-related arousals they also provide the information needed to diagnose conditions such as RBD and bruxism, as well as provide other information that can support or confirm a specific finding other than what they are traditionally used for.

It is important to keep in mind that the electrodes in the submental area simultaneously record the EMG activity of the upper airway dilator as well as other cervical muscles, movement artifacts, EKG, and even EEG. Special skills and experience are needed for the accurate interpretation of a polysomnogram and taking advantage of subtle findings can help in this regard.

## Conclusions

Recording electrical activity from the submental area provides the essential information needed for the interpretation of PSG. It is recommended to expand the HSAT montage to include an additional frontal electrode to record EEG and one to monitor activity in the submental area.
